# Multi-gene targeted antiangiogenic therapies for experimental corneal neovascularization

**Published:** 2010-02-27

**Authors:** Peng Chen, Hongmei Yin, Yao Wang, Jing Mi, Wenxiao He, Lixin Xie, Yiqiang Wang

**Affiliations:** 1 Shandong Provincial Key Lab of Ophthalmology, Shandong Eye Institute, Qingdao, China; 2 Department of Surgery, Duke University Medical Center, Durham, NC

## Abstract

**Purpose:**

To determine the effectiveness of multigene-based anti-angiogenic gene therapies for experimental murine corneal neovascularization (corneal NV).

**Methods:**

Recombinant retroviral vectors encoding murine endostatin (mEndo), murine-soluble vascular endothelial growth factor receptor-2 (msFlk-1), or murine-soluble Tie2 (msTie2) were constructed and packaged in PT67 cells. Viral titers were determined by infection of NIH3T3 cells. Expressions of mEndo, msFlk-1, and msTie2 were confirmed by reverse transcription PCR. The 3-(4,5-Dimethyl-2-thiazolyl-2,5-diphenyl-2H-tetrazolium bromide (MTT) assay was used to estimate the effect of mEndo, msFlk-1, or msTie2 on the proliferation of human umbilical vein endothelial cells, and the scarification test was used to measure the migration of the cells. Seventy C57Bl/6 mice were subjected to the induction of chemical-burn corneal NV and tested for efficacy of gene therapy. Gene therapy was performed by subconjunctival injection of viral preparations and its effect was evaluated by scoring corneal NV.

**Results:**

The recombinant virus-producing cell lines expressing mEndo, msFlk-1, and msTie2 were constructed successfully. Overexpression of these putative anti-angiogenic proteins inhibited the proliferation and migration of human umbilical vein endothelial cells in vitro. In the murine corneal NV model, subconjunctival injection of the retroviral particles of mEndo and msFlk-1 showed the most significant inhibition of corneal NV.

**Conclusions:**

Gene therapy with the recombinant retroviral vector-hosted mEndo and msFlk-1 gene effectively inhibited corneal NV induced by alkaline burn. The combination of multiple anti-angiogenic genes might be necessary for effective therapy of corneal NV, although each of these pathways makes a potential target for the treatment of this disease.

## Introduction

Neovascularization is a complex process and is tightly regulated by many positive and negative factors [1-3]. It plays important roles in several diseases of the eye, resulting in vision impairment or blindness. The cornea is normally avascular to permit optimal visual clarity; neovascularization, however, can occur in pathologic conditions. Corneal neovascularization (corneal NV) is a central feature in the pathogenesis of many blinding corneal disorders and is also a major sight-threatening complication in corneal infections and chemical injury or after keratoplasty. Effective and practical approaches to diminishing or completely preventing corneal NV remain to be found. Anti-angiogenic therapy may serve as a strategy for such ocular disease therapy. Since increased secretion of vascular endothelial growth factor (VEGF) [4-6] has been observed in corneal NV as well as in other ocular neovascularization, some of the efforts have been directed at blocking VEGF or its endothelial cell-specific receptors, namely vascular endothelial growth factor receptor 1 (Flt-1) and vascular endothelial growth factor receptor 2 (Flk-1). Thus VEGF-targeted neutralizing antibodies, antisense oligonucleotides, soluble receptors, or nuclease-resistant aptamers have been reported to inhibit neovascularization in laboratory research or clinical practice [7-11].

Activation of nuclear factor-κB (NF-κB) reportedly leads to the expression of VEGF [12-14]. Deletion of NF-κB binding sites within the VEGF promoter abolishes VEGF expression in many cells, suggesting that activation of NF-κB is essential for VEGF upward regulation induced by various stimuli [15]. Other angiogenesis or anti-angiogenesis pathways are also important in the development of neovascularization. For example, endostatin (Endo) is an endogenous inhibitor of angiogenesis [16]. Systemic administration of Endo by gene delivery resulted in reduced corneal NV during 36 days posttransplantation [17]. The angiopoietin(Ang)–Tie2 system in endothelial cells also participates in vasculogenesis and maintenance of vascular integrity. Ang1 and Ang2 have been identified as ligands of the endothelial cell-specific Tie2 receptor [18,19]. Targeted gene inactivation in vivo or transgenic overexpression studies suggest that Ang1 recruits and sustains periendothelial support cells, while Ang2 disrupts blood vessel formation in the developing embryo by antagonizing the effects of Ang1 on Tie2 [19,20]. Targeting the Ang–Tie2 pathway with soluble Tie2 (sTie2) has been shown to block tumor angiogenesis [21], and nuclease-resistant RNA aptamer specific for Ang2 also inhibits rat corneal neovascularization [22].

Although successful anti-angiogenic therapies have been demonstrated in animal neovascularization models with certain factors, systematic comparisons of the potency of different anti-angiogenic factors in corneal NV situations have not been described. While therapy targeting a single anti-angiogenic gene cannot block corneal NV development completely [23], we tried to explore optimal combinations of multigene-based anti-angiogenic therapies for corneal NV. In this study, we investigated the anti-angiogenic activity of strategies targeting VEGF, Tie2, and Endo to inhibit alkaline burn-induced corneal NV in mice.

## Methods

### Generation of retroviral particles

The retroviral expression vector used in this study was pMSCV (BD BioSciences Clontech, San Jose, CA), a Moloney murine leukemia-virus-derived retroviral vector capable of prolonged transgene expression in vivo through a modified long-terminal repeat (LTR). The DNA fragments of murine endostatin (mEndo), murine-soluble vascular endothelial growth factor receptor 2 (msFlk-1) and murine-soluble Tie2 (msTie2) were generated by polymerase chain reaction (PCR). Recombinant retroviral vectors hosting mEndo, msFlk-1, or msTie2 were constructed as described previously [24] and transfected into PT67 cells, using Effectence Transfection Reagent (Qiagen GmbH, Hilden, Germany). Transfected PT67 cells were selected against puromycine (2 μg/ml), and the stable transfectants were expanded and named vehicle vector-PT67, mEndo-PT67, msFlk-1-PT67, and msTie2-PT67. Virus-containing culture supernatants were collected and filtered through a 0.45-μM filter unit. Viral titers were determined by infection of NIH3T3 cells, as described by others [25] and adjusted to 5×10^6^ plaque-forming units (pfu)/ml. NIH3T3 cells were plated at 2.5×10^5^ in a 60 mm dish on the previous day. To determine viral titer, serially diluted viral supernatants were added to NIH3T3 in the presence of 8 μg/ml polybrene. The next day, transduced cells were transferred to 100 mm plates and selected in the presence of puromycine until visible colonies were formed. Viral titer was determined by counting the number of drug-resistant colonies. The retrovirus was purified by using ViraTrapTM retrovirus purification kit (Biomiga, San Diego, CA) as follows: One to two T75 flasks of the retrovirus-infected culture media were centrifuged  at 1,000× g for 10 min. The column was equilibrated with 2 ml of double-distilled water and then with 5 ml of 1× kit wash buffer. The supernatant was loaded onto the column and allowed to gradually run through. The flow through was collected and reloaded to the same column one more time to ensure maximal viral particle binding. The column was then washed, and the retrovirus was eluted. The titer of the concentrated viral stock was 1×10^7^ pfu/ml. The virus was stored at −70 °C for further use.

### Infection of human umbilical vein endothelial cells and detection of proliferation and migration

Human umbilical vein endothelial cells (HUVEC) were seeded in 96-well plates at 2,500 cells per well in 100 μl of DMEM (Gibco, Grand Island, NY) complete medium (plus 10% fetal calf serum) 1 day before transfection. Viral supernatant containing vehicle vector-pMSCV, mEndo-pMSCV, msFlk-1-pMSCV, or msTie2-pMSCV was added to the culture to 1×10^6^ pfu/well and allowed to grow 24 h before being replaced with 100 μl of prewarmed fresh medium supplemented with 10 μl of 5 mg/ml 3-(4,5-Dimethyl-2-thiazolyl-2,5-diphenyl-2H-tetrazolium bromide (MTT). The contents of each retroviral particle in every group were as follows: vehicle vector, 1×10^6 ^pfu of vehicle retroviral particles; msFlk-1, 1×10^6 ^pfu of msFlk-1 retroviral particles; msTie2, 1×10^6^ pfu of msTie2 retroviral particles; mEndo, 1×10^6 ^pfu of mEndo retroviral particles; msFlk-1/msTie2, 5×10^5 ^pfu of msFlk-1 retroviral particles and 5×10^5 ^pfu of msTie2 retroviral particles; msFlk-1/mEndo, 5×10^5 ^pfu of msFlk-1 retroviral particles and 5×10^5 ^pfu of mEndo retroviral particles; msTie2/mEndo, 5×10^5 ^pfu of msTie2 retroviral particles and 5×10^5 ^pfu of mEndo retroviral particles; msFlk-1/msTie2/mEndo, 3.3×10^5 ^pfu of msFlk-1 retroviral particles, 3.3×10^5 ^pfu of msTie2 retroviral particles, and 3.3×10^5 ^pfu of mEndo retroviral particles. Four hours later the medium was removed and the cells dissolved in 100 μl of DMSO. Absorbances were measured using a MultiSkan plate reader (Molecular Devices, Sunnyvale, CA) at a wavelength of 492 nm.

For the measurement of cell migration, a wound-healing assay was used [26]. Briefly, HUVEC cells were seeded on 24-well tissue culture plates. When the culture reached 90% confluence, a wound was made with a micropipette tip in the center of the culture plates. The cultures were rinsed with culture medium to remove detached cells and incubated with medium containing viral supernatant from vehicle vector-PT67, mEndo-PT67, msFlk-1-PT67, or msTie2-PT67 cells for another 16 h. Digital images of wound closure were obtained and used for quantitative assessment of migration by measuring the distance of cells that migrated beyond the original injury borders. Each assay was conducted at least in triplicate.

### Induction of cornea neovascularization and therapy

All animal experiments were performed in accordance with the guidelines of the Association for Research in Vision and Ophthalmology Statement for the Use of Animals in Ophthalmic and Vision Research. Seventy C57B1/6 mice of both sexes (male:female=1:1), 8–10–weeks old, were purchased from the Institution of Laboratory Animal Sciences, Chinese Academy of Medical Sciences (Beijing, China) and were housed at the Shandong Eye Institute Animal Facility. Briefly, the mice were anesthetized intraperitoneally with ketamine (37.5 mg/ml) and xylazine (1.9 mg/ml). Proparacaine hydrochloride (0.5%) was used for topical anesthesia. For induction of corneal NV, a piece of disk-shaped filter paper 2.0 mm in diameter was immersed in 1 mol/l NaOH solution for 15 s and placed on the central corneal surface for 50 s to produce a circular burn, followed by immediate washing with 30 ml of 0.9% saline. Only the right eye of each mouse was used for corneal NV induction; the left eye was undisturbed. For gene therapy, viral preparations were injected into the subconjunctival site on the same day of the corneal NV induction. Briefly, a needle pierced into the conjunctiva from the corneoscleral limbus, and 7 μl of purified retroviral particles (1×10^7^ pfu/ml) or saline was injected. Thirty-six hours following the operation, another 7 μl of retroviral particles was administrated into the conjunctival sac. The contents of each retroviral particle in every group were as follows: normal group, untreated; saline group, 7 μl of 0.9% saline; vehicle vector, 7 μl of vehicle retroviral particles; msFlk-1/msTie2, 3.5 μl of msFlk-1 retroviral particles and 3.5 μl of msTie2 retroviral particles; msFlk-1/mEndo, 3.5 μl of msFlk-1 retroviral particles and 3.5 μl of mEndo retroviral particles; msTie2/mEndo, 3.5 μl of msTie2 retroviral particles and 3.5 μl of mEndo retroviral particles; msFlk-1/msTie2/mEndo, 2.3 μl of msFlk-1 retroviral particles, 2.3 μl of msTie2 retroviral particles, and 2.3 μl of mEndo retroviral particles. Each group contained ten C57B1/6 mice. Eyes were examined daily under a slit-lamp biomicroscope (Zeiss, Jena, Germany) for 7 days from the alkaline burn. Cornea photographs were taken with a camera mounted on the slit-lamp microscope under the same magnification. Neovascularization of each cornea, as described by others [27], was determined by a blind examiner to minimize the observer’s bias. Briefly, the neovascularization score=(distance from the limbus to the end point of the cornea neovascularization/distance from the limbus to burn)/0.17. Each cornea received an exact score. Scores were concluded representatively as follows: 0 (no visible vessels in the cornea), +1.5 (1/4 distance to burn), +2 (1/3 distance to burn), +3 (1/2 distance to burn), +4 (2/3 distance to burn), +4.5 (3/4 distance to burn), and +6 (vessels reach burn).

### Reverse-transcription PCR

Total RNA was prepared from each cornea, using the NucleospinRNA kits (BD Biosciences, Palo Alto, CA), and reverse transcribed into first-strand cDNA, using Primescript™ First-Strand cDNA Synthesis kit (TaKaRa, Dalian, China). Each group contained three corneal tissues. Gene-specific cDNA fragments were amplified with DNA polymerase (Tiangen, Beijing, China), using the following primers: matrix metalloproteinase-9 (*MMP-9*) forward 5′-CGT CGT GAT CCC CAC TTA CTA-3′ and reverse 5′-AAG ATG AAC GGG AAC ACA CAG-3′; interleukine-1β (*IL-1β*) forward 5′-CAG AGG ATA CCA CTC CCA ACA-3′ and reverse 5′-TCC AGT TTG GTA GCA TCC ATC-3′; glyceraldehyde-3-phosphate dehydrogenase (*GAPDH*) forward 5′-GGG CAC CGA TGG CGT TGA GT-3′ and reverse 5′-GCT GTG GTG GGG GCT GTG GA-3′. PCR amplification products were analyzed by agarose gel electrophoresis.

### Antibodies and western blotting

Total protein was prepared from each cornea, using radio immunoprecipitation assay (RIPA) buffer (50 mmol/l Tris pH 7.4, 150 mmol/l NaCl, 1% Triton X-100, 1% sodium deoxycholate, 0.1% sodium dodecyl sulfate [SDS], sodium orthovanadate, and sodium fluoride; Galen, Beijing, China), and quantified. Protein (50 µg in 15 μl loading buffer) was resolved in 10% sodium dodecyl sulfate polyacrylamide gel electrophoresis (SDS–PAGE) gel and then transferred to a polyvinylidene difluoride membrane (Millipore, Billerica, MA). The blots were blocked in 5% nonfat dry milk dissolved in Tris-buffered saline Tween-20 (TBST; 20 mmol/l Tris, pH 7.5, 0.5 mmol/l NaCl, 0.05% Tween-20) for 1 h and then incubated with the primary antibody in TBST for 1 h, followed by incubation with horseradish peroxidase-conjugated secondary antibody (Amersham Biosciences, Uppsala, Sweden) for 1 h. All incubations were done at 25 °C, and three washes with 10 ml TBST were applied between each step. The membranes were then developed with SuperSignal West Femto Maximum Sensitivity substrate (Pierce Biotechnology, Rockford, IL) and exposed to X-ray film (Kodak, Rochester, NY). The bands were analyzed using National Institutes of Health (NIH) Image 1.62 software (NIH, Bethesda, MD). Primary antibodies included anti-VEGF antibody (Abcam, Cambridge, MA), anti-nuclear factor of kappa light polypeptide gene enhancer in B-cells inhibitor alpha (IκBα) antibody (Boster, Wuhan, China), anti-GAPDH antibody (Kangchen, Shanghai, China), anti-phosphorylated nuclear factor-kappa B (P-NF-κB) antibody, and anti-NF-κB antibody (Cell Signaling Technology, Danvers, MA). All the experiments reported in this study were performed three times, and the results were reproducible. Immunoreactive bands were quantified by using NIH Image 1.62. For each sample, the levels of proteins of interest were normalized to that of GAPDH.

### Inflammatory cells infiltration in corneas

Eyes were enucleated 7 days after the alkaline burn, fixed in 10% buffered formalin, and embedded in paraffin. Paraffin sections of 4 μm in thickness were deparaffinized, rehydrated, and stained with hematoxylin and eosin staining. Three eyes were included in each group. To count inflammatory cells, three fields of each paraffin section in each group close to the axis running from the center of new capillaries to the center of the cornea were randomly selected, and the total number of leukocytes was counted under a microscope in a blind fashion. The total cell counts in each slide were averaged, and an analysis of variance (ANOVA) was carried out.

### Statistical analysis

Data are presented as mean±standard deviation (SD). The differences between control and experimental conditions were evaluated by SPSS 11.5 software (SPSS Inc., Chicago, IL), and p<0.05 was considered significant.

## Results

### Anti-angiogenic genes inhibit proliferation and migration of endothelial cells

**Figure 1:**
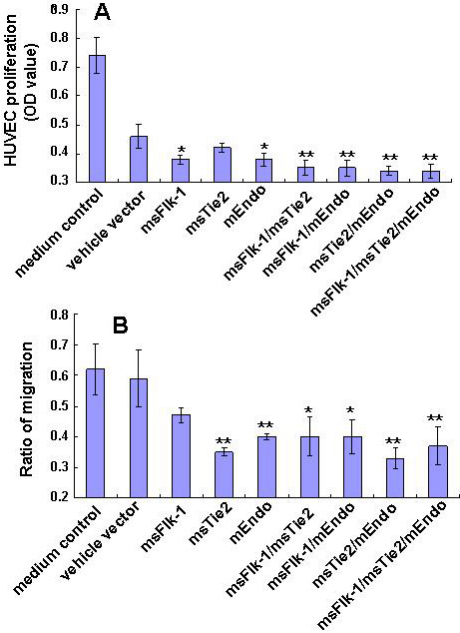
Effect of murine endostatin (mEndo), murine-soluble vascular endothelial growth factor receptor 2 (msFlk-1), and murine-soluble Tie2 (msTie2) expression on proliferation and migration of HUVEC cells. **A**: Equal numbers of HUVEC cells were incubated for 24 h in viral supernatant from vehicle vector-PT67, mEndo-PT67, msFlk-1-PT67, or msTie2-PT67 cells. The viable cell number was measured by the conversion during 4 h of MTT into soluble formazan. Bars represent standard deviation (SD; n=3 wells per measurement). Similar results were obtained in three independent experiments. **B**: HUVEC cells were seeded on 24-well tissue culture plates. When the cells reached 90% confluence, a wound was made with a micropipette tip in the center of the culture plates. The cultures were rinsed to remove detached cells and incubated with medium containing viral supernatant from vehicle vector-PT67, mEndo-PT67, msFlk-1-PT67, or msTie2-PT67 cells for 16 h. Digital images of wound closure were captured and used for quantitative assessment of migration by measuring the distance cells migrated beyond the injury lines. Four independent experiments were conducted, and data were shown as mean±SD. The asterisk indicates p<0.05, and the double asterisk indicates p<0.01, compared with vehicle vector cells.

We first measured the effects of viral preparations on proliferation and migration of HUVEC cells. It was not surprising, although unexpected, that infection of HUVEC with the vehicle vector construct alone inhibited HUVEC proliferation significantly since the “control vector efficacy” phenomenon had been described in other systems as well [28,29]. Except for msTie2, all other gene constructs, alone or in combination, inhibited HUVEC proliferation further when compared to the vehicle vector (Figure 1A). Migration of HUVEC cells was significantly inhibited by transfection with mEndo, msFlk-1, msTie2, and their combinations, as determined by wound-healing assays (Figure 1B). While the combination of multiple genes showed significant additive or synergistic effects over single genes in the proliferation assay (Figure 1A), such effectd were not observed in the migration assay (Figure 1B). The inhibitory effect of the vehicle vector observed in the HUVEC proliferation assay was not observed in the migration assay either.

### Prevention of neovascularization in the cornea

**Figure 2:**
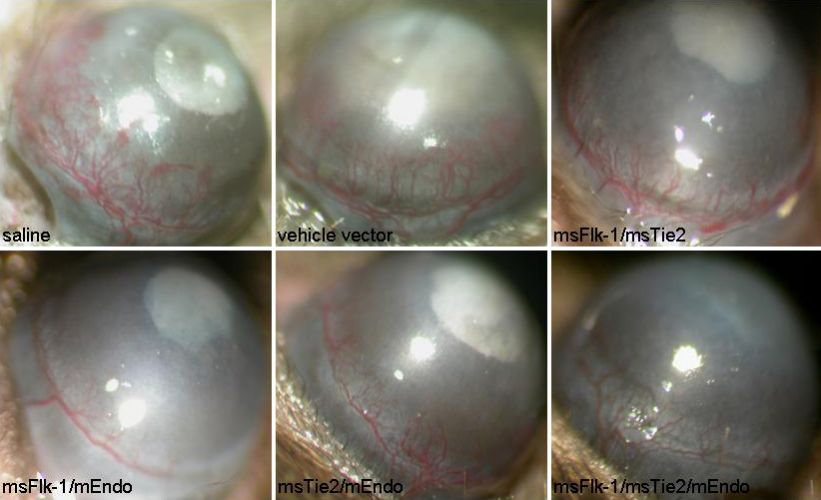
Effectiveness of multigene-based anti-angiogenic gene therapies for experimental murine corneal neovascularization (corneal NV). Zones of inhibition of neovascularization were visible in corneas injected with retroviral virus expressing mEndo, msFlk-1, msTie2, and their combinations.

We next studied the effects of mEndo, msFlk-1, or msTie2 gene combinations on alkaline-induced corneal NV. At day 7 after corneal NV induction and the start of gene therapy, the inhibitory effect of combined mEndo, msFlk-1, or msTie2 gene therapy was obvious when compared to eyes treated with the vehicle vector or saline (Figure 2, Table 1). Meanwhile, the number or density of load vessels in combined gene-treated eyes was less than in controls (Figure 2).

**Table 1:** Inhibition of corneal NV by retroviral virus expressing msFlk-1, msTie2, or mEndo (mean±SD).

**Groups**	**Number of eyes**	**Neovascularization score**			**p (versus saline)**	**p (versus vehicle vector)**
** **	** **	**Maximum**	**Minimum**	**Mean**	** **	** **
Saline	10	3.5	1	2.17±0.74	-	-
vehicle vector	9	3	1.5	2.16±0.35	p>0.05	-
msFlk-1/msTie2	10	2.5	0.5	1.6±0.49	p<0.01	p<0.01
msFlk-1/mEndo	10	2.5	0.5	1.27±0.71	p<0.01	p<0.01
msTie2/mEndo	10	3.25	0.75	1.51 ±0.53	p<0.01	p<0.01
msFlk-1/msTie2/mEndo	10	2.8	0.5	1.41±0.54	p<0.01	p<0.01

Neovascularization was scored according to the distance from the limbus to the farthest end of the new vesicles: 0 (no visible vessels in cornea), +1.5 (1/4 distance to burn), +2 (1/3 distance to burn), +3 (1/2 distance to burn), +4 (2/3 distance to burn), +4.5 (3/4 distance to burn) and +6 (vessels reach burn).

### Inflammatory cell infiltration during the angiogenic process

**Figure 3:**
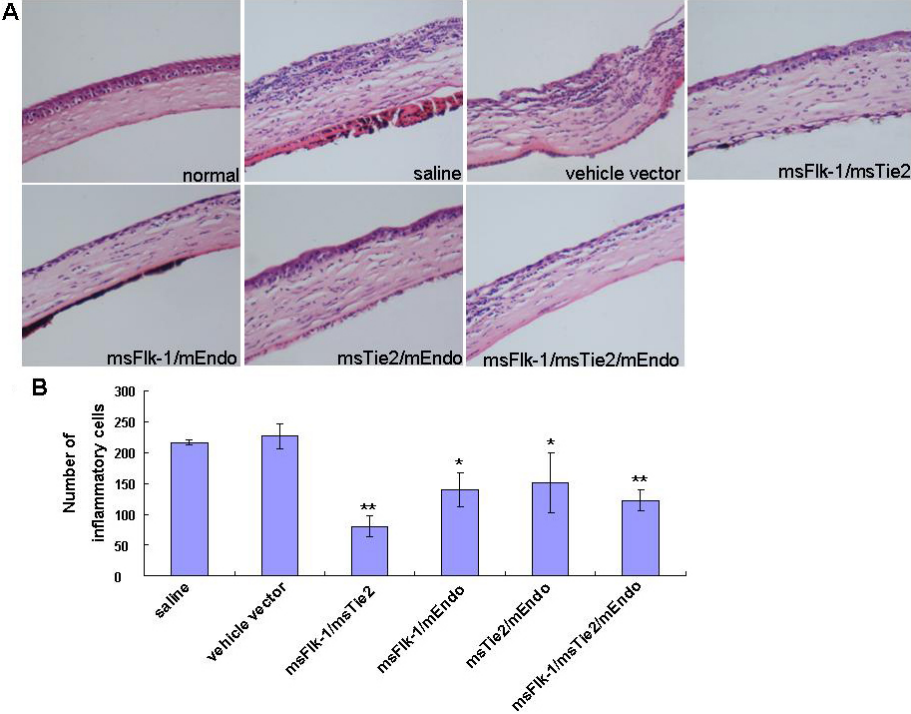
Combined gene therapy groups had significantly fewer inflammatory cell infiltrates. **A**: Histology of chemically burned corneas stained with hematoxylin and eosin (200×). **B**: Three fields of each slide close to the axis running from the center of new capillaries to the center of the cornea were selected, and the total number of inflammatory cells was counted under a microscope in a blind fashion. The total cell counts in each slide were averaged and an analysis of variance (ANOVA) was carried out. Three sections of each group were observed and shown as mean±SD. The asterisk indicates p<0.05, and the double asterisk indicates p<0.01, compared with the vehicle vector group.

In corneal diseases, several cytokines and growth factors are upregulated and induce infiltration of neutrophils, macrophages, and lymphocytes [30]. Infiltration of inflammatory cells is often accompanied by an angiogenic response [31]. Therefore, we analyzed the infiltration of inflammatory cells in this model. Histological studies revealed that corneas in all combined gene therapy groups had significantly fewer inflammatory cell infiltrates than did corneas in the saline and vehicle vector groups (Figure 3).

### Effect of gene therapy on expression of angiogenic factors and nuclear factor-κB pathway activity in alkaline-burned corneas

**Figure 4:**
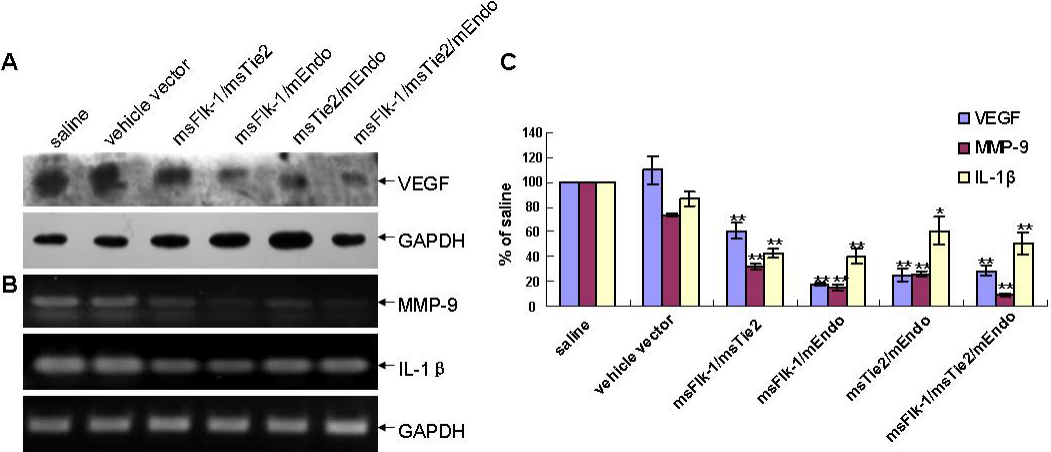
Effects of gene therapy on vascular endothelial growth factor (VEGF), matrix metalloproteinase-9 (MMP9), and interleukine-1β (IL-1β) expression in alkaline-burned corneas. VEGF was detected by western blot (**A**) while *MMP9* and *IL-1β* were detected by reverse transcription PCR (**B**). GAPDH was used as the internal control in all cases. As shown by the analysis results (**C**) compared with the vehicle vector group, combined gene therapy significantly downregulated the expression levels of *VEGF*, *MMP-9*, and *IL-1β*. Three independent experiments were conducted, and data were shown as mean±SD. The asterisk indicates p<0.05, and the double asterisk indicates p<0.01, compared with the group injected vehicle retroviral particles into conjunctival sac (vehicle vector group).

**Figure 5:**
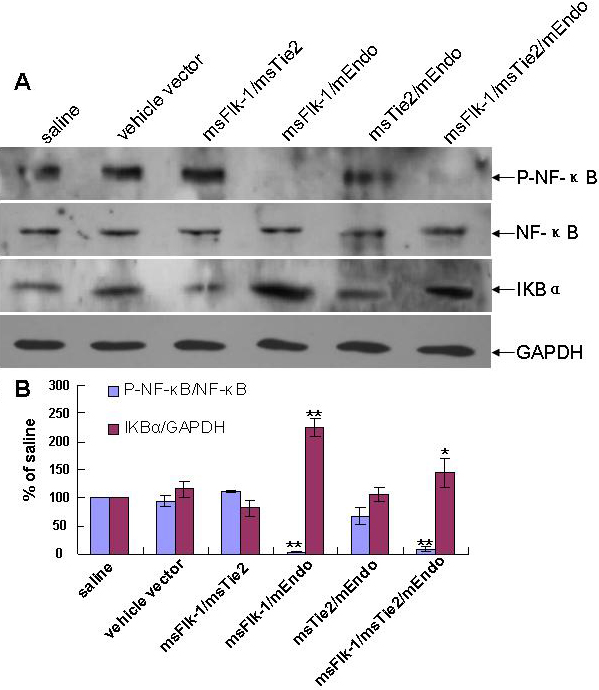
Measurement of nuclear factor-kappa B (NF-κB) pathway proteins by western blotting in alkaline-burned corneas. **A**: Total lysates from murine corneal tissue lysates were analyzed by western blotting for their level of NF-κB activation by detecting phosphorylated NF-κB (P-NF-κB), total NF-κB protein, and IκBα. As shown by the analysis results (**B**), msFlk-1/mEndo and msFlk-1/msTie2/mEndo significantly downregulated NF-κB phosphorylation expression. Three independent experiments were conducted, and data were shown as mean±SD. The asterisk indicates p<0.05, and the double asterisk indicates p<0.01, compared with the vehicle vector group.

Expression of VEGF or MMP-9 and IL-1β in corneas at day 7 after corneal NV induction was measured by using western blotting or RT-PCR. We found that the combination of mEndo, msFlk-1, and msTie2 genes effectively inhibited VEGF, MMP-9, and IL-1β expression in the alkaline-burned corneal NV model (Figure 4). Lastly we examined activity of the NF-κB pathway and found that msFlk-1/mEndo and msFlk-1/msTie2/mEndo significantly reduced NF-κB phosphorylation and IkBα expression without changing the total amount of NF-κB (Figure 5).

## Discussion

Due to its noninvasive accessibility and clarity, cornea serves an ideal target for evaluating the efficacy of various treatments against corneal disorders. A multitude of potential gene therapies based on different genes and strategies have been tried in the context of corneal diseases, including corneal NV. For example, various viral-mediated gene delivery systems have been proposed to produce expression of the desired genes with varying expression levels and duration [32-34].

In the case of gene therapy for corneal NV, as in many other situations, it is well recognized that targeting a single gene often fails to ensure the theoretically predicted efficacy [23,35-37]. We therefore investigated the possibility of better management of corneal NV by combining different anti-angiogenic factors in the present study. We showed that the combination of two or three genes exhibited additive effects over a single gene in inhibiting HUVEC proliferation in vitro (Figure 1A). This additive effect, however, was not observed when HUVEC migration was used as the end point (Figure 1B), confirming that different pathways might be involved in proliferation or migration processes of endothelial cells in response to the same stimulus [38,39].

Using an inflammation-associated corneal NV model, we further proved that targeting neovascularization with such a combination of genes effectively inhibited neovascularization in the corneas. Western blotting and RT-PCR assay showed that such anti-angiogenic activity might involve a decrease in VEGF secretion and IL-1β/MMP-9 expression. As a principal degrader of extracellular matrix (ECM), MMP-9 was proposed to facilitate the growth of new blood vessels by breaking down the ECM and amplifying effects of other angiogenic factors [40], thus representing a logical target for anti-angiogenic therapy. IL-1β is one of the key mediators involved in many inflammatory responses, including chemical-burn-induced corneal NV [41-43]. In the current study, although without direct evidence, we assume that the observed decrease in neutrophil infiltration in corneas of gene therapy groups might be due to the decrease of IL-1β expression in corneas.

On the other hand, NF-κB plays an important role in IL-1β-related inflammatory diseases, including various corneal diseases [44,45]. In the corneal alkali burn model, NF-κB is activated in the corneal epithelial and stromal cells [44]. NF-κB is an important therapeutic target in chronic inflammatory diseases, enabling significant downregulation of macrophage-produced pro-inflammatory cytokines [46-48]. NF-κB is normally sequestered in the cytoplasm by IκB. NF-κB activation requires phosphorylation, induced ubiquitination, and degradation of cytoplasmic IκB. There are conflicting data in the literature regarding the role of NF-κB in VEGF signaling. One group has been unable to demonstrate an effect of the NF-κB pathway on the expression of VEGF [49]. Others have shown that the upregulation expression of VEGF is regulated by increased NF-κB activity [50,51]. Signaling via the NF-κB pathway has recently been suggested to play a role in the transcriptional regulation of the *VEGF* gene in various cancer cells [52,53]. In addition, administration of NF-κB antisense oligonucleotides partially inhibited the tumor necrosis factor-α and platelet-activating factor-dependent production of VEGF in human vascular endothelial cells [54].

In summary, our study directly confirmed that combinations of mEndo, msFlk-1, and msTie2 may be promising anti-angiogenic alternatives for treatment of corneal diseases with neovascularization. Preliminary findings also supported a functional role for NF-κB in the VEGF signaling pathway, but more in-depth studies should be carried out to expound the exact mechanisms for such gene therapy.
